# Remedies and Their Uses

**Published:** 1907-04-13

**Authors:** 


### Remedies and their Uses.

### Veronal.

This body belongs to the now rather extensive class of synthetic preparations derived from urea, and it is not a little curious that the advance of science should have placed bodies of an excrementitious nature again on the list of remedial agents, whence they had been so long excluded. Veronal is di-ethyl-malonyl-urea, or diethyl barbituric acid

$$\begin{matrix}C_2H_5\\C_2H_3\end{matrix}\!\!>\!C\!<\!\!\begin{matrix}CO\ HN\\CO\ HN\end{matrix}\!\!>\!CO.$$

and is a white crystalline powder, of slightly bitter taste, soluble in 145 parts of water at ordinary temperatures, and in 12 parts of boiling water. It is not very soluble in cold alcohol or in chloroform, but is easily soluble in hot alcohol and ether. It is chemically a fairly stable resistant substance, and is not easily decomposed. Physiologically, experiment has shown that veronal is by no means a highly toxic substance; it has no action on the blood corpuscles or hæmoglobin, and may be injected intravenously in solution. When given in solid form to warm-blooded animals by the mouth, it is fairly rapidly dissolved in the alkaline intestinal contents and is absorbed, but the action is not so powerful as when a solution is given in the first instance. It is excreted fairly rapidly in the urine, by far the major part without chemical change. Large, toxic doses cause depression of the higher centres and collapse; traces of albumen appear in the urine, and in cases of chronic poisoning there is considerable loss of weight.

For the rest, the important points in the pharmacology of veronal are: (1) it has no action on the heart or blood vessels, and may thus be safely given in cardiac disease; (2) although it is a diuretic, this property is not marked when the drug is given in ordinary medicinal doses; it has no irritative action on the kidneys; (3) it is said to have some slight power of sparing proteid, and would thus be useful in wasting diseases; (4) its comparatively slow absorption and easy elimination render the risk of toxic symptoms from an overdose slight and unimportant.

Veronal is given usually in doses of 8-12 grains, though smaller doses (4 or 5 grains) may be enough. It is best given dissolved in hot milk, tea, or alcohol. The sleep produced by veronal is very like natural sleep; it may be expected from half an hour to an hour after the administration of the drug, and lasts several hours. According to the experience of all observers, veronal is most successful in cases of simple insomnia not accompanied by actual pain, or at any rate not accompanied by pain of any severe type. In various mental disorders, melancholia mania, dementia, paranoia, it has been largely and successfully employed; in epileptic and hysterical conditions it is of value, and also in neurasthenia and hypochondriasis. In the treatment of morphinism and alcoholism it will be found efficacious even for bad cases, as also for delirium tremens, the insomnia of pyrexia, and for severe hysteria and epilepsy; doses of 15-25 grains may be required.

Subacute uræmia and dyspepsia may also be safely treated with veronal, owing to its non-irritant nature.

It may also be given to children, to which class of patients its comparatively slight taste will recommend it. Infants can take $\frac{3}{5}$-$1\frac{1}{4}$ grains once or twice a day.

Veronal may be given combined with antipyrin, which is well tolerated by children, as a remedy for whooping cough. The following formula is recommended:

| | |
|---|---|
| Veronal ... ... ... ... ... | gr. 15 |
| Antipyrin ... ... ... ... ... | gr. 15 |
| Syr. zingiber. ... ... ... ... | ℥j. |
| Aq. ad ... ... ... ... ... | ℥iv. |

F.M.

*Sig.*: One teaspoonful four times daily.

With older children (above three years) more veronal may be given (up to 30-35 grains). If necessary, veronal may be given as an enema, as it is easily dissolved and readily absorbed from the intestine.

In severe cough and intense pain veronal fails to act, and in certain cases of arteriosclerosis, also in old people its action is occasionally much delayed. In chronic cases of insomnia a slight cumulative action has been noticed, so that it is not necessary to give it every night. In the night sweats of phthisis, owing to the slight diuretic action, veronal is said to be extremely useful. Doses as small as 5 grains may be efficacious, but the effect will not be noted for a night or two.

In cases where there is much pain some anodyne preparation, not in itself a hypnotic, may be combined with veronal. Aspirin in doses of 10-15 grains may be used, or methyl atropine bromide ($\frac{1}{15}$ grain) may thus be employed.

Idiosyncrasy against veronal does not appear to be common. The following symptoms have, however, been noted: giddiness, mental confusion, vomiting, scarlabiniform or morbilliform rashes, marked diuresis. A single large dose of 45 grains taken by an hysterical girl produced deep sleep with convulsions and feeble cardiac action, requiring stimulants. Under the second heading may be mentioned one fatal case in which half-an-ounce was consumed in about forty-eight hours. In another case in which severe symptoms occurred the patient (a lunatic) eventually recovered. She appears to have taken a dose of 9 grams (135 grains), apparently in the solid form. She became comatose in the afternoon, and her stomach was washed out. She remained comatose until the third day, when she was merely very drowsy; on the fourth day her mind was clear. The other symptoms were slight stertor, general relaxation of the muscles, with occasional convulsions resembling those of tetanus but much slower, and a bullous eruption. The pulse and respiration remained good, but later on the latter became very superficial; the reflexes were practically unaffected. There was some opisthotomes.

